# Global perspectives on food fraud: results from a WHO survey of members of the International Food Safety Authorities Network (INFOSAN)

**DOI:** 10.1038/s41538-019-0044-x

**Published:** 2019-07-17

**Authors:** John Spink, Peter Ben Embarek, Carmen Joseph Savelli, Adam Bradshaw

**Affiliations:** 1 College of Veterinary Medicine, Michigan State University, 1129 Farm Lane, East Lansing, MI 48864 USA; 2 International Food Safety Authorities Network (INFOSAN) Management, Department of Food Safety and Zoonoses, World Health Organization (WHO), 20, Avenue Appia, 1211 Geneva 27, Switzerland; 3 Department of Food Safety and Zoonoses, World Health Organization, Geneva, Switzerland

**Keywords:** Economics, Risk factors

## Abstract

This survey of International Food Safety Authorities Network (INFOSAN) members regarding food fraud prevention, management, education, and information sharing included 166 WHO member states that resulted in 175 responses. The respondents engage in food fraud prevention (70%) or are responsible for food fraud incident response (74%). Nearly all respondents acknowledged a desire for more guidance and information on best practices in managing the full range of “food safety events involving food fraud” (97%), but also for prevention of such events (97%), indicating a need to provide technical support beyond acute incident response. The scope of food fraud covered in the survey comprised the full range of fraudulent activities, including the addition of adulterant-substances, tampering (including mislabeling), theft, smuggling, gray market/diversion, and counterfeiting (intellectual property rights). Key needs included: capacity-building/education; a platform for information sharing; and utilization of INFOSAN as an interagency/intergovernmental collaboration point.

## WHO survey: Understand the perspectives of INFOSAN members on food fraud

Food fraud risk is an intentional act driven by economical profits and not intended to pose a public health threat like food terrorism. Spink from Michigan State University and coworkers reported the results of a WHO food fraud survey of members of the International Food Safety Authorities Network (INFOSAN). Most of these INFOSAN members are involved in food fraud prevention or food fraud incident response in their agencies/organizations. The respondents consider the food fraud activities or prevention programs to include adulterant-substances, tampering, counterfeits, theft, smuggling, and gray market/diversion. This study revealed their concerns about food safety events involving food fraud and the needs of educating, managing, and preventing fraud related food safety events. These understandings are important for the further considerations of next steps.

## Introduction

The International Food Safety Authorities Network (INFOSAN) is a global network of national food safety authorities, managed jointly by the Food and Agricultural Organization of the United Nations (FAO) and the World Health Organization (WHO). INFOSAN provides an important platform for the rapid exchange of information during food safety emergencies and for sharing data and information pertaining to routine and emerging food safety issues.^1^ INFOSAN members were asked to participate in a survey to better assess the current understanding of the issues related to food fraud globally. The survey was distributed only to the official key contact for each member state and to encourage open feedback the surveys were anonymous. Future more formal priority setting and financial allocation would include official and formal responses. Specifically, the survey explores the level of activity related to managing food safety issues involving food fraud prevention, the level of concern and the challenges each respondent’s organization faces in managing fraud-related food safety issues.

Administration of this survey was conducted in follow-up to a technical meeting organized by the Nanyang Technological University of Singapore in November 2016 for INFOSAN members in Asia, as well as food safety regulators, academics, and laboratory scientists from around the world. Previously, the INFOSAN Secretariat at WHO was asked by INFOSAN members to provide more information and resources on food fraud.^2^ In response, the first action was to present and discuss food fraud at the meeting in Singapore. During the meeting, discussion on food fraud focused on sharing experiences and information on food fraud events and the increased importance of responding to intentional contamination and other fraudulent practices that can result in food safety emergencies. The discussion also considered how INFOSAN could best be utilized to support members in managing these issues, and the possible development of resources to help members share information and build capacity.

During the November 2016 meeting, there was debate and discussion about the needs of the member states and also whether INFOSAN should include food fraud issues at all. The important first step is to assess the interest and needs of the member states before INFOSAN more formally considers next steps.

This survey intended to elicit a better global understanding of this issue from the INFOSAN-member perspective.

Food Fraud—illegal deception for economic gain using food which includes the US-centric subcategory of economically motivated adulteration (EMA)—is rising in awareness and concern.^3–11^ Serious public health consequences to instances of food fraud have emphasized the need for coordinated action in order to mitigate negative impacts. Incidents such as Sudan Red dye in ground chili, melamine in infant formula and pet food, horsemeat in beef products, peanut allergen in cumin, waste oil in cooking oil, and others—all demanded complex international reactions and illustrated vulnerabilities in food regulatory systems around the world.^12,13^

Food fraud can be understood as one category within the food risk continuum which also includes food quality, food safety, and food defense. These categories represent different types of food risks that cover intentional and unintentional acts as well as incidents that cause public health harm and others that do not. The types of food risks are defined to focus on the motivating factors in order to support prevention activities.^14^ These include:^3^

- Food quality risk: an unintentional act that results in a food product not meeting the stated or required attributes or standards;
- Food safety risk: an unintentional act that results in a food product that poses a health concern if consumed as intended;
- Food fraud risk: an intentional act on a food product that is economically motivated and not intended to pose a public health threat; and
- Food defense risk: an intentional act on a food product that is intended to pose a public health threat, such as malicious tampering or terrorism.

Other than food defense, these general definitions are consistent with those used in texts developed by the WHO/FAO Codex Alimentarius Commission (CAC). Food Defense is referred to, but not defined, within such texts.

**Fig. 1:**
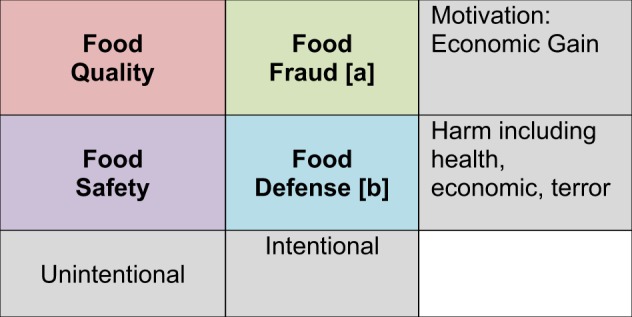
The Food Risk Matrix [3]: **a** Includes the subcategory of economically motivated adulteration or EMA. **b** Includes acts of terrorism

The food risk matrix has been used to explain the differences between the food risks^3^ (Fig. 1).

While the vast majority of food fraud incidents do NOT cause a public health threat, the lack of oversight, controls, or confirmation of ‘good manufacturing practices’ creates a system vulnerability. Food fraud risks can be further characterized as:^3^

- Direct food risk: occurs when the consumer is put at immediate or imminent risk, such as the inclusion of an acutely toxic or lethal contaminant, that is, one exposure can cause adverse effects in the whole or a smaller at-risk population.
- Indirect food risk: occurs when the consumer is put at risk through long-term exposure, such as the buildup of a chronically toxic contaminant in the body, through the ingestion of low doses. This risk also includes the omission of beneficial ingredients, such as preservatives or vitamins.
- Technical food risk: is nonmaterial in nature. For example, food documentation fraud occurs when product content or country-of-origin information is deliberately misrepresented.

The laws, regulations, standards, certifications, and best practices for food fraud prevention are just developing. Work on the development of methods and the administration of tests related to food authenticity has a long history and is now expanding in order to consider a range of food fraud types. Other supply chain or traceability programs are also being expanded to meet additional requirements for food fraud prevention. This recent attention has led to food fraud being addressed by governments in the USA, the UK, China, and Europe, (e.g., US Food Safety Modernization Act, UK National Food Crime Unit, Chinese Food Safety Law, European Commission Food Integrity Project, etc.) and by others commercial entities as well (e.g., Global Food Safety Initiative GFSI, ISO Technical Committee 292 Security Management/Work Group 4 on Product Fraud, ISO Technical Committee 34/Sub-Committee 17 on Food Supply Chain Management, and others).^9,10,15^

Concerns have been expressed at the CAC regarding food fraud that has led the Codex Committee on Food Import and Export Inspection and Certification Systems (CCFICS) in 2017 to create an Electronic Working Group (EWG) to draft a food fraud/food integrity/food authenticity discussion paper.^16^ The CCFICS provided clear directions to address broad fraud types rather than only adulterant-substances and to focus on prevention rather than only authenticity test methods.^17,18^ The EWG is tasked to review; (1) definitions of the terms; and (2) current gaps in the Codex Alimentarius. One important issue is that the Codex Alimentarius has published a definition of “contaminant” which is an ingredient that is unintentionally added and included at a level that is unacceptable,^19^ but there is no corresponding definition of “adulterant” (adulterant-substance).

In 2016, the CAC updated the document titled “Principles and Guidelines for the Exchange of Information in Food Safety Emergency Situations (CAC/GL 19-1995),” that included appropriate references to INFOSAN. Launched in 2004, INFOSAN facilitates the sharing of food safety information and experience and promotes collaboration between food safety authorities at national and international levels, especially during emergencies. INFOSAN members include government nominated representatives from national agencies responsible for food safety emergency response or another aspect of national food safety.

## Results

The survey results are separated into sections including: demographics, general questions, types of food fraud, and recommendations or open comments.

### Demographics

The respondents included members of the INFOSAN Advisory Group (3%), INFOSAN Emergency Contact Points (26%), INFOSAN Focal Points (52%), and others (19%) from within national agencies responsible for food safety emergency response or another aspect of national food safety management (Table 1). A portion of the respondents (7%) attended the November 2016 INFOSAN meeting that presented the food fraud prevention topics. Of the 453 recipients, there were 185 survey responses with 136 completed responses.

**Table 1:** Demographics of the survey respondents (of 183 responses)

Demographics					
1	INFOSAN Advisory Group	INFOSAN Emergency Contact Point	INFOSAN Focal Point	Other (please specify)	Skipped
	3%	26%	52%	19%	6
2	Did you attend the INFOSAN conference November 7–8, 2016 in Singapore where food fraud was presented?				
	Yes	No	Skipped		
	7%	93%	5		

### General questions

The general questions were presented in clusters related to specific topics (Table 2).

**Table 2:** General questions (of 183 responses)

General questions					
	Yes	No	Don’t know	Prefer not to answer	Skipped
3	Does your agency/organization currently engage in any form of food fraud prevention activities?				
	70%	27%	2%	1%	43
4	Does your agency/organization manage or respond to any form of food fraud events?				
	74%	17%	3%	1%	39
5	Has your organization conducted a Food Fraud Vulnerability Assessment?				
	13%	73%	11%	3%	51
6	If yes, was the data your organization found and used adequate for your purpose? (You did not need to seek more or different data)?				
	28%	26%	28%	21%	122
7	Do you consider Food Fraud a “Food Safety” issue? (E.g. an issue related to food safety controls and thus an issue to be addressed by your food safety system?)				
	93%	6%	0%	0%	56
8	In your country is food fraud dealt with by an agency/authority not dealing with food safety? (E.g. customs, law enforcement, etc.)				
	40%	44%	11%	5%	49
9	When an international event involving food fraud occurs and no food safety concerns are identified, should INFOSAN play a role in sharing this information as it does with international food safety events?				
	75%	14%	9%	2%	52
10	Would your agency/organization be willing to share information related to food fraud events through INFOSAN?				
	69%	4%	20%	6%	57
11	Would you like to receive more guidance and information on best practices in managing food safety events involving food fraud?				
	97%	1%	2%	0%	48
12	Would you like to receive more guidance and information on best practices in preventing food safety events involving food fraud?				
	97%	1%	2%	1%	50

**Addresses food fraud prevention**—**questions 3**–**4:** The respondents’ organizations are engaged in food fraud prevention (70%), and they do manage or respond to food fraud incidents (74%). There is clarity on the direct assignment of the roles and responsibilities (demonstrated by the low 3% who stated the “Did not know” or “Prefer not to answer”). Thus, it appears that broad bases of INFOSAN members are engaged with food fraud incident prevention and management.

**Vulnerability assessments and datasets**—**question 5**–**6:** While the respondents’ organizations respond to incidents they do not conduct proactive food fraud vulnerability assessments (FFVA) or risk assessments (84% “No” or “Don’t know”). Of those who did conduct FFVAs, most did not find complete incident datasets (55% said “No” or “Don’t know”). Thus, the need is both to raise awareness of the capabilities and limitations of datasets collected as part of FFVAs as well as to improve capacity to conduct incident reviews.

**Food safety and INFOSAN**—**question 7**–**10:** While food fraud is overwhelmingly considered a food safety issue by respondents (93%) the authority responsible for responding to or addressing food fraud is often not in a food safety agency (e.g., customs, law enforcement, etc.) (55% said “No” or “Don’t know”). The respondents do indicate that INFOSAN plays an important role in addressing food fraud (75% “Yes” to 14% “No”) and suggest they would share information on food fraud (69% “Yes” to 4% “No” and more ambiguity with 20% “Don’t know”).

**Support for the member states**—**question 11**–**12:** Beyond the provision of assistance during an acute food fraud incident, there was an overwhelming indication that respondents would like to receive more guidance and information on best practices in managing (97%) and preventing (97%) “food safety events involving food fraud.” There is an unmet need for capacity building in the prevention and management of food fraud-related events as well as a need for education on the different types of fraud and the ways to detect and respond to them.

### Types of food fraud

INFOSAN members were questioned about the ‘Types of Food Fraud’ in order to better understand the scope of food fraud activities that are addressed by the INFOSAN members (Table 3). To keep the survey short, and increase the response rate, the definitions proposed were not covered in more detail at this stage. Beyond the traditional adulterant-substances (73%), tampering (57%), and counterfeits (39%) focus there was awareness of the other types of fraud (theft 16%, smuggling 27%, and gray market/diversion 27%) (Note: for this survey mislabeling was included in tampering). The INFOSAN members are involved in a broad range of food fraud types. There should be further review of the tampering and adulterant types to clarify whether this includes malicious acts which would be categorized as food defense. There is a need for further examination of the definition and scope of national food fraud prevention activities in different countries to define the unmet needs for support and capacity building.

**Table 3:** Food fraud types (of 183 responses)

Food fraud types		
13	What does your agency/organization consider to be the scope of fraud currently addressed by its food fraud activities or prevention program? (Select all that apply):	
	Responses	Answer choices
	73%	Adulterant-substances (adulterants)
	57%	Tampering (including mislabeling
	16%	Theft
	27%	Smuggling
	27%	Gray market/diversion
	39%	Counterfeiting/intellectual property rights
	9%	Don’t know
	4%	Prefer not to answer
		Other (please specify)
	Skipped	49

### Respondent recommendations and open comments

Finally, an open question was included, seeking additional ideas, concerns, and suggestions (Table 4). Among the comments, there were requests for more information and for INFOSAN to be a collaboration point. The 47 comments provided did cluster into several main categories including capacity building/education (22), a platform for information sharing (10), and for INFOSAN as an interagency/intergovernmental collaboration point (11). Overall, results have identified an unmet need among INFOSAN members for support with a focus on exchanging information on food fraud issues that have a food safety component.

**Table 4:** Selection of open comments or recommendations that apply to the research question

Selection of open comments or recommendations		
1	Fraud is in its nature a criminal act and should be handled by authorities. Cases concerning fraud are handled very differently from food safety cases. Fraud cases are mostly handled by law enforcement or authority in cooperation with law enforcement and require special permission for information to be shared. Food fraud is no exception hence information that can be shared through INFOSAN in real time is very limited and often of no use to other parties.	
2	INFOSAN should send alerts only for food frauds that may have food safety concerns. However, food control authorities in the member countries should have measures in place to ensure that food frauds are addressed, as in this informal (not regulated) hence, may cause harm to consumers.	
3	It is important now to not set up too many different systems, obliging us to report, and follow-up. The CODEX work on identifying existing texts related to fraud should be a priority for the international cooperation and any involvement of INFOSAN should be related to either international agreements on what needs to be shared and how. Sharing such information not related to immediate health risks may compromise ongoing investigations into bigger fraud networks.	
4	It is necessary to improve communication with each other and increase cooperation opportunities.	
5	It would be important for INFOSAN to train its members in an accurate understanding of the meaning of food fraud and its scope, so it would also be important to instruct them on the management to be carried out when food fraud is detected in domestic and imported products.	
6	Keep alert the epidemiological surveillance and share information. Also provide training regarding food safety to the personal	
7	Scope should be limited to food safety concerns only	
8	The role of coordination between the various systems could be useful in the global market	
9	Timely sharing of information if there is a food safety issue involved	
10	To continue strengthening the network on information sharing to create a transparent global food safety platform by inviting more members	

This preliminary study provides a baseline for understanding the concerns and needs of some INFOSAN members.

## Discussion

This survey established and clarified the INFOSAN member concerns about food fraud and the unmet need for help to educate, manage, and prevent fraud-related food safety events. Potential immediate next steps may include: (1) development of a Food Fraud Fact Sheet with dissemination to INFOSAN members; (2) presentation of resources for education and capacity building to INFOSAN members; and (3) development and administration of a more detailed and targeted survey to better understand the issue at the individual country level.

Respondents from INFOSAN are addressing food fraud prevention are seeking both guidance and leadership on managing incidents and on overall food fraud prevention. It is clear that the resources and support system for addressing food fraud prevention is complex and will be different from many of the current food safety activities. The interdisciplinary activities expand from traditional food science and food safety to include international supply chain management, social science, criminology, forensic accounting, intelligence analysis, and other trade or customs tactics. The “effect” is often a public health food safety incident, but the “cause” is often a fundamentally different *modus operandi*. Several large scale food fraud incidents have required active and sustained coordination through INFOSAN, so there is already a precedent and a need to exchange information on food fraud issues that present a food safety risk. There is also an opportunity to share information in advance of an explicit food safety issue to shift towards a proactive rather than a reactive system of response.

As the food fraud vulnerability becomes clearer, and the unmet needs of the member states, the further research is needed on the holistic, all-encompassing, and global approach to reducing the fraud opportunity, protecting the food supply chain, and strengthening food security. Further research could include sharing of best practices, consideration of new innovations or technologies, information sharing, and alert systems, and others approaches and perception about food fraud and food authentication.

## Methods

The development of an online, anonymous food fraud survey was led by the INFOSAN Secretariat at WHO and was administered and analyzed by the Michigan State University Food Fraud Initiative. To stimulate more open and direct comments, no personal identifiers were used, so the survey is anonymous. One drawback of the anonymous process is that the responses represent the opinions of individual INFOSAN members and not individual countries or the WHO Member States. As such, the total number of countries represented by the responses could not be confirmed. Solicitation of a formal response by country can be conducted in a later phase. The survey was conducted from May 2017 to September 2017. The INFOSAN Secretariat distributed it in English, French, and Spanish to 453 INFOSAN members from 166 WHO the Member States, six areas/territories of the WHO Member States and two associate members. This survey conforms to the Michigan State University Institutional Review Board policies. The respondents signaled their compliance by accepting the first survey question.

## Data Availability

The datasets generated during and/or analyzed during the current study are available from the corresponding author on reasonable request.
